# Illness prevalence rate in Tibet, China: data from the 2018 National Health Service Survey

**DOI:** 10.1186/s12889-020-08960-7

**Published:** 2020-06-18

**Authors:** Rendan Deng, Labasangzhu, Zhaxideji, Guihua Wang, Peiwei Hong, Jiaqi Li, Qiwen Li, Yang Wan, Hai Xiong

**Affiliations:** 1 West China School of Public Health and West China Fourth Hospital, Sichuan University, Chengdu, 610041 China; 2 Medical College of Tibet University, Lhasa, 850000 China

**Keywords:** Tibetan, Two-week prevalence rate, Factors

## Abstract

**Background:**

Tibet is located in the high-altitude area of Southwest China, where the health level is influenced by specific factors such as the natural environment and living habits. However, there has been little research that has focused on Tibetan health conditions. The two-week prevalence rate is an important indicator of the health level of residents. The purpose of this study was to understand the health status of the residents and the health service needs in Tibet.

**Methods:**

The two-week prevalence rate was calculated using data from a population of 10,493 individuals aged 15 and above that was obtained from the 2018 Sixth National Health Service Survey of Tibet. We initially analysed the types and associated factors of two-week illnesses in Tibetan. The influencing factors for the two-week prevalence rate in Tibet were determined by multivariate logistic regression analysis. Subsequently, we assessed the severity of two-week illnesses by calculating the average days of the duration of the disease, the days of being bedridden and the days of being off work.

**Results:**

The two-week illness prevalence rate was 20.1% in Tibet. Digestive system diseases were frequent, and hypertension was the most common disease. According to the multivariate logistic regression analysis, the two-week prevalence rate was associated with gender, age, residence, marital status, and employment status. In addition, the severity of two-week illnesses differed among the residents.

**Conclusion:**

This study identified that health service needs have increased in Tibet and that the health status of the local residents needs to be improved. Moreover, hypertension has become a major health hazard for the residents and should be considered in the utilization of health services.

## Background

The Tibet Autonomous Region is located in Southwestern China. It covers an area of 1.23 million square kilometres and has a population of approximately 3.3 million. The natural environment and living habits of Tibetans are significantly different from those in other parts of China, as the altitude is over 4000 m [1]. The local residents mainly obtain nutrients and energy from traditional foods such as Zanba, Tibetan salt cream tea, and Tibetan milk tea [2, 3]. Many countries, such as the United States, Ethiopia and Kenya, attach importance to local health services to establish effective health service systems [4–6]. In addition, China has been developing a primary health-care system [7].

Previous studies have shown that morbidity is the dominant predictor of health service utilization. In addition, the two-week prevalence rate is an important index for evaluating health service needs, which can reflect the health level and social health status of the population [8]. In China, there are huge differences in the two-week prevalence among different regions [9, 10]. The Fourth National Health Service Survey of China showed that Chengguan District in Lahsa, Tibet has a minimal two-week prevalence rate of 5.2%, while the Dongcheng District in Beijing has a maximal two-week prevalence rate of 53.2% [11]. The Fifth National Health Service Survey of China showed that the two-week prevalence rate was 32.1% in West China, which is lower than the 26.2% prevalence rate found in East China [12]. The health status of the local residents in Tibet has seldom been reported. In this study, the two-week prevalence rate and factors based on data from the Sixth National Health Service Survey of Tibet, China, which was completed in 2018, are reported.

## Methods

### Data source

Data were obtained from the 2018 Sixth National Health Service Survey of Tibet, China. The Tibet Autonomous Region has jurisdiction over seven prefecture-level cities, consisting of 74 counties. Therefore, according to the level of economic development, geographical location, population distribution and other factors, 13,102 residents from 3060 households were finally included in this survey, according to the multistage stratified cluster random sampling method.

By performing a face-to-face survey using an electronic tablet, the investigator inquired about all the members of each household one by one, filled in the related electronic questionnaires offline [13], and then uploaded the survey data online after the survey instructors examined the responses for each person. Because this study was a national survey project and was organized by the relevant departments of the government, the selected residents actively cooperated with the survey. Therefore, there was no refusal to answer. However, there were 525 cases with missing data. Subjects were eligible to participate in the current study if they (1) were ≥ 15 years old and (2) were permanent residents of the sample households. A total of 10,493 valid cases were finally included. In principle, all the contents of the survey should be answered by the respondents. However, people who were not at home or those who were unable to respond during the survey period were replaced by those who were familiar with their situation.

This study was part of the Sixth National Health Service Survey of China, which was approved by the National Health and Family Planning Commission of the People’s Republic of China and by the Health and Family Planning Commission of the Tibet Autonomous Region. Oral consent was obtained before the eligible residents took the survey.

### The definitions of the outcome variables

The illness types are listed in Additional file 1. A two-week illness was defined as the respondents having undergone any of the following three circumstances less than 2 weeks before being interviewed: 1) visiting a doctor; 2) receiving medical treatment for the illness or injury; or 3) being bedridden or off work due to illness (including obvious abnormal depression and loss of appetite in elderly people) for at least 1 day.

The two-week prevalence rate was calculated by the following formula: Two-week prevalence rate = (Number of respondents with two-week illness) * 100% / (The total number of respondents).

Gender, age, residence, education, economic level, marital status, and employment status were selected as covariates to examine their impacts on the two-week prevalence rate. We divided age into 4 groups: 15–29 years old, 30–44 years old, 45–59 years old, and 60- years old. Residence was divided into 2 groups: rural and urban. Education was divided into 5 groups: illiterate, primary school, junior middle school, high school, and university and above. Economic level was grouped according to the quartile of annual income per capita: low, medium and high, with the grouping cut-offs being 3333 yuan, 6000 yuan, and 12,000 yuan, respectively. Marital status was divided into married, unmarried, widowed, divorced and other. Employment status was divided into employed, retired, laid-off, unemployed, and student. In addition, we measured the severity of the two-week illness by calculating the average duration of the disease in days, the number of days of being bedridden and the number of days of being off work.

### Statistical analysis

A chi-square test was performed to examine the significance of the differences in the two-week prevalence rates according to the demographic variables. Whether an individual was sick within the previous 2 weeks was used as a dichotomous variable. A multivariate logistic regression analysis was further conducted, and the variables with statistical significance were included in the analysis. With respect to the number of subjects suffering from chronic diseases, a multivariate regression analysis was performed to adjust for confounding factors, including gender, age, residence, economic level, education level, economic level, marital status, and employment status. In addition, a one-way analysis of variance was used to determine the severity of the two-week prevalence rate in different groups. The data analysis was completed using Statistical Package for Social Science (*SPSS*) version 20.0 statistical software (IBM, Armonk, NY, USA) and *p* < 0.05 was set as the test level.

## Results

Overall, 10,493 residents aged 15 and above were included in this study. The proportion of women (53.1%) was higher than that of men. The average age of the subjects in this study was 44.1 ± 15.7 years old. Tibetans accounted for the highest proportion (97.0%) among the ethnic groups in Tibet. The two-week prevalence rate was 20.1% in Tibet in 2018 (see Table 1).

**Table 1:** Sociodemographic characteristics and illness prevalence rates among residents over 15 years old from Tibet in 2018

Characteristics		Number	Proportion (%)	Two-week Illness		*p* value
				Number of illnesses	Prevalence rate (%)	
	Total	10,493	100.0	2104	20.1	
Gender	Male	4921	46.9	795	16.2	< 0.001
	Female	5572	53.1	1309	23.5	
Age	15–29	2086	19.9	176	8.4	< 0.001
	30–44	3412	32.5	511	15.0	
	45–59	3245	30.9	865	26.6	
	60-	1750	16.7	553	31.6	
Residence	Urban	2186	20.8	606	27.7	< 0.001
	Rural	8307	79.1	1498	18.0	
Education	Illiterate	5037	48.0	1181	23.4	< 0.001
	Primary school	3496	33.3	668	19.1	
	Junior middle school	1217	11.6	156	12.8	
	High school	603	5.7	90	14.9	
	University and above	140	1.3	10	7.1	
Economic level	Low	2524	24.1	457	18.1	0.008
	Medium	5311	50.6	1075	20.2	
	High	2658	25.3	572	21.5	
Marital status	Married	7950	75.7	1624	20.4	< 0.001
	Unmarried	1567	15.0	148	9.4	
	Widowed	719	6.9	258	35.8	
	Divorced	186	1.8	59	31.6	
	Others	71	0.7	16	22.5	
Employment status	Employed	8232	78.4	1483	18.0	< 0.001
	Retired	207	2.0	77	37.2	
	Laid-off	168	1.6	70	41.7	
	Unemployed	1601	15.3	467	29.1	
	Student	285	2.7	8	2.8	

Digestive diseases, cardiovascular diseases, musculoskeletal diseases, respiratory diseases, and urogenital diseases accounted for the top five diseases by body system, with prevalences of 27.8, 20.5, 16.5, 11.6 and 4.4%, respectively. Moreover, in terms of the composition of diseases, the top five chronic diseases were hypertension (14.4%), rheumatoid arthritis (8.0%), cholelithiasis (6.3%), chronic gastritis (5.5%), and diabetes mellitus (0.9%) (data not shown).

Among the patients, the two-week prevalence rate of females was significantly higher than that of males. The two-week prevalence rate was positively associated with age and economic level, and it was inversely associated with education. Urban residents had a higher two-week prevalence rate than that of rural residents (27.7% vs. 18.0%). The two-week prevalence rate of widows was the highest, reaching 35.8%. Among people with different employment statuses, the two-week prevalence rate of the unemployed population was the highest, reaching 41.7%, followed by that of the retired population at 37.2%. The characteristics of the survey participants are presented in Table 1.

To further study the influencing factors of the two-week prevalence rate, a regression analysis was performed. A chi-square test found that the two-week prevalence rate was associated with gender, age, residence, economic level, marital status and employment status. In an unadjusted regression analysis, the participants who were women, older, and urban residents and those who had a higher economic level and poor marital and employment statuses had a higher odds ratio (OR) for morbidity compared with the other groups. Other than for economic level, the effect size remained significant after adjusting for other factors (see Table 2).

**Table 2:** The influencing factors of the two-week prevalence rate according to the univariate and multivariate analysis of individuals from Tibet in 2018

Influence factor		Crude OR	95% CI	Adjusted OR	95% CI	^&^*p* value
Gender	Female	1.000		1.000		
	Male	0.627	(0.569,0.692)	0.691	(0.622,0.767)	< 0.001
Age	15–29	1.000		1.000		
	30–44	1.912	(1.595,2.290)	1.464	(1.201,1.784)	< 0.001
	45–59	3.944	(3.318,4.689)	2.804	(2.304,3.413)	< 0.001
	60-	5.000	(4.158,6.013)	2.968	(2.367,3.722)	< 0.001
Residence	Urban	1.000		1.000		
	Rural	0.574	(0.514,0.640)	0.610	(0.541,0.689)	< 0.001
Education	Illiterate	1.000		1.000		
	Primary school	0.772	(0.694,0.859)	0.921	(0.822,1.032)	0.155
	Junior middle school	0.481	(0.401,0.576)	0.876	(0.717,1.072)	0.199
	High school	0.573	(0.454,0.724)	0.974	(0.741,1.279)	0.849
	University and above	0.251	(0.132,0.480)	0.568	(0.288,1.120)	0.102
Economic level	Low	1.000		1.000		
	Medium	1.148	(1.017,1.296)	1.134	(1.000,1.287)	0.051
	High	1.240	(1.081,1.423)	1.106	(0.953,1.283)	0.185
Marital status	Married	1.000		1.000		
	Unmarried	0.407	(0.340,0.486)	0.684	(0.562,0.832)	< 0.001
	Widow	2.182	(1.856,2.565)	1.279	(1.070,1.529)	0.007
	Divorce	1.781	(1.324,2.478)	1.644	(1.187,2.277)	0.003
	Others	1.134	(0.648,1.984)	1.023	(0.575,1.819)	0.940
Employment status	Employed	1.000		1.000		
	Retired	2.696	(2.022,3.593)	1.295	(0.945,1.776)	0.108
	Laid-off	3.251	(2.380,4.440)	2.360	(1.695,3.287)	< 0.001
	Unemployed	1.868	(1.655,2.110)	1.238	(1.075,1.424)	0.003
	Student	0.131	(0.065,0.266)	0.334	(0.159,0.701)	0.004

&: *p* value adjusted by multivariate logistic regression analysis

/: In multivariate regression analysis, the Enter method was used to adjust for confounding factors

In addition, the duration of the two-week illness was positively associated with age. There were differences according to urban-rural residence, education, annual income per capita, marital status, and employment status. The duration of being bedridden for the two-week illness also differed by age, residence, education, and employment status. Rural residents were off work longer than urban residents. (see Table 3).

**Table 3:** The severity of two-week illnesses in Tibet in 2018

Characteristics		^a^ Duration		^a^ Being bedridden		^a^ Being off work	
		Mean	95% CI	Mean	95% CI	Mean	95% CI
Gender	Male	8.47	(8.13,8.82)	4.53	(3.51,5.54)	1.64	(0.18,3.10)
	Female	8.54	(8.27,8.81)	4.43	(3.78,5.07)	2.29	(0.03,4.56)
*ǂ Age	15–29	7.41	(6.72,8.10)	3.04	(1.54,4.55)	1.67	(0.21,5.46)
	30–44	7.77	(7.36,8.19)	3.40	(2.56,4.25)	2.20	(1.77,6.17)
	45–59	8.52	(8.19,8.86)	4.05	(3.19,4.91)	1.94	(0.08,3.80)
	60-	9.54	(9.14,9.95)	6.32	(5.14,7.50)	2.17	(0.34,7.74)
*#ǂ Residence	Urban	8.18	(7.77,8.59)	4.20	(3.24,5.16)	1.40	(0.12,2.92)
	Rural	8.65	(8.41,8.89)	4.59	(3.93,5.25)	2.29	(0.38,4.19)
*ǂ Education	Illiterate	9.05	(8.78,9.33)	5.35	(4.55,6.15)	2.58	(0.68,5.85)
	Primary school	7.89	(7.51,8.26)	3.55	(2.74,4.36)	1.53	(0.09,2.98)
	Junior middle school	7.54	(6.76,8.32)	2.36	(0.76,3.96)	2.00	(0.70,3.30)
	High school	8.00	(6.89,9.11)	4.31	(1.95,6.67)	/	/
	University and above	7.00	(2.93,11.07)	/	/	/	/
*Economic level	Low	8.45	(8.01,8.89)	3.93	(2.91,4.94)	4.62	(3.36,5.88)
	Medium	8.67	(8.38,8.96)	4.92	(4.09,5.75)	2.83	(2.07,3.60)
	High	8.28	(7.86,8.70)	4.13	(3.12,5.14)	2.38	(1.43,3.33)
*Marital status	Married	8.33	(8.09,8.56)	3.98	(3.39,4.56)	1.84	(0.48,3.20)
	Unmarried	9.10	(8.29,9.91)	4.77	(2.61,6.94)	1.00	(0.12,13.71)
	Widow	9.31	(8.69,9.93)	6.54	(4.70,8.38)	6.50	(0.76,8.90)
	Divorce	8.10	(6.82,9.39)	4.11	(0.54,7.68)	/	/
	Others	10.94	(8.66,13.22)	6.00	(1.93,10.07)	/	/
*ǂ Employment status	Employed	8.11	(7.86,8.36)	3.61	(3.02,2.00)	2.00	(0.67,3.33)
	Retired	9.74	(8.56,10.92)	3.33	(0.13,6.79)	/	/
	Laid-off	10.00	(8.87,11.13)	6.53	(3.25,9.81)	/	/
	Unemployed	9.40	(8.95,9.85)	6.17	(5.03,7.32)	/	/
	Student	7.63	(3.29,11.96)	/	/	/	/

^a^: measured in days

*: There were differences in duration among the different groups, *p* < 0.05

ǂ: There were differences in being bedridden among the different groups, *p* < 0.05

#: There were differences in the duration of being off work among the different groups, *p* < 0.05

/: There were no subjects satisfying the grouping condition

## Discussion

Based on the 2018 Sixth National Health Service Survey in the Tibet Autonomous Region, the two-week prevalence rate and its influencing factors were analysed. This study showed that the two-week prevalence rate of the residents aged 15 years and older in Tibet was 20.1% in 2018, which was higher than the rate from the Fifth National Health Service Survey in Tibet (10.6%) [12]; this finding indicates that the health service needs in Tibet significantly increased between the times of the two surveys. The two-week prevalence rate is an important index for evaluating the utilization of health services. Our results showed that the two-week prevalence rate in Tibet was influenced by multiple factors. On the one hand, health insurance coverage has increased from 29.7% in 2003 to 97.0% in 2015 in China. Health insurance coverage has reached approximately 95% in Tibet [14, 15]. Additionally, both the education level and health awareness of Tibetans have improved. Therefore, more residents choose to actively seek medical treatment.

Among the disease systems, the digestive system had the largest proportion of diseases; however, cardiovascular system diseases were prioritized according to the national survey results [12] and may be related to food habits (such as special Tibetan dietary habits). The traditional Tibetan diet is almost 60% protein and high in fat [2]. In the traditional Tibetan dietary model, protein and fat provide approximately 60% of the daily energy intake, making food difficult to digest and be absorbed by the human body. Moreover, hypertension has been reported as the most common chronic disease in Tibet, which may be related to the diet and awareness of Tibetans [16, 17]. A high-salt diet and an insufficient awareness of hypertension lead to an increase in medical treatment [18, 19]. Nevertheless, in this study, the self-reported prevalence of hypertension was 14.4% among the patients, which was lower than the national level (23.2%) [20]. This outcome indicates the inadequate utilization of health services to a certain extent in Tibet.

In this study, we found that the two-week prevalence rate among females was higher than that among males. The reason for this outcome may be that women have special physiological periods, namely menstruation, pregnancy, childbirth, puerperium and breastfeeding, which result in special needs [21, 22]. Compared with men, women have lower immunity and more delicate emotions, making them more likely to pay attention to their own health needs. According to the findings of Anna Ruggieri [23], it appears that differences in hormonal, genetic and environmental factors between males and females may affect immune responses. In addition, females may more actively utilize health services because they pay more attention to their health than do males.

With increasing age, the two-week prevalence rate showed a linearly increasing trend, which is supported by studies [24] that have shown that various kinds of physical diseases gradually increase with increasing age. For most older people, physical and social activities show a downward trend, which weakens the immunity of the body. In addition, most women over 60 are menopausal, and their health might thus be affected by hormone levels [25]. Older people who are more sensitive to illness may promote their use of medical services.

In addition, we found that the two-week prevalence rate may be related to residence, marital status, and employment status. We identified factors that differed between rural and urban residences of two-week prevalence rates. The risk of the two-week disease of urban residents was higher than that of rural residents, which may be because the education level and health awareness of urban residents were higher than those of farmers and herdsmen [26, 27]. Moreover, the distance between residential areas and clinical areas in agricultural and pastoral areas was relatively farther than that in urban areas, which might affect the accessibility of medical treatment for farmers and herdsmen to a certain extent. Therefore, the reported prevalence rate was low, which was similar to the results from Tian, D [28]. Compared with married people, the two-week prevalence of widowed and divorced people was higher. The reason may be that the previous way of life or environment of people who experienced widowhood or divorce, to a certain extent, would be changed. On the other hand, widowed patients were also more likely to be older, and the results were consistent with age. Therefore, widowed patients may experience a certain negative psychological impact on their health. A happy marriage and good family care are conducive to reducing the occurrence of illness and accelerating one’s recovery from illness. In different employment situations, unemployment and being laid off were risk factors for a two-week illness, which is similar to the results from the Fifth National Health Service Survey [12]. To a certain extent, an irregular daily life and realistic pressure are negative factors of illness [29]. As a special social group, school students are at an early life stage and have relatively low life pressure and regular living habits, and most of them are energetic because of their youth and have good physical immunity; thus, their possibility of experiencing a two-week illness was low.

There were several limitations in this study. First, this cross-sectional study was insufficient for making causal inferences. Therefore, we could only provide the possible influencing factors for the two-week illness rate. Second, because the respondent’s illness rate was self-reported, the actual two-week illness rate may be underestimated due to recall bias and a low diagnosis rate. Third, due to the lack of longitudinal data, we were unable to examine changes in the two-week prevalence rate.

## Conclusion

In conclusion, the two-week prevalence rate in 2018 was significantly higher than that from the Fifth National Health Survey in Tibet, indicating that the needs for health care have increased greatly and that the health level needs improvement. The two-week prevalence rate of Tibetans was generally associated with gender, age, residence, marital status, and employment status. In addition, the severity of the two-week prevalence rate was different among groups based on age, residence, education, marital status, and employment status. Therefore, many efforts should be made by the central and local governments of China to improve the health of Tibetans because of the severity of the disparity. This study may provide a basis for formulating health service policies about residents with different characteristics for the government.

## Supplementary information

**Additional file 1.** The classifications of diseases in the Sixth National Health Service Survey, 2018.

## Data Availability

The datasets that support the findings of this study are available from Medical College of Tibet University but are restricted to the availability of these data, which were used under license for the current study, and so are not publicly available. Data are however available from the authors upon reasonable request and with permission of Medical College of Tibet University.

## Abbreviations

CI: Confidence interval
OR: Odds ratio

## References

[1] Shen L, Zhang X, Dawawuzhu (2019). Pain Clinic in Tibet, China: a single-center retrospective study. Pain Res Manag.

[2] Wang Z, Dang S, Xing Y (2017). Dietary patterns and their associations with energy, nutrient intake and socioeconomic factors in rural lactating mothers in Tibet. Asia Pac J Clin Nutr.

[3] Sánchez-Villegas A, Delgado-Rodríguez M, Martínez-González MA (2003). Gender, age, socio-demographic and lifestyle factors associated with major dietary patterns in the Spanish project SUN (Seguimiento Universidad de Navarra). Eur J Clin Nutr.

[4] Blackwell DL, Lucas JW, Clarke TC. Summary health statistics for U.S. adults: national health interview survey, 2012. Vital Health Stat 10. 2014;(260):1–161. https://pubmed.ncbi.nlm.nih.gov/24819891/. PMID: 24819891. 24819891

[5] Girma F, Jira C, Girma B (2011). Health services utilization and associated factors in Jimma zone, south West Ethiopia. Ethiop J Health Sci.

[6] Ngugi AK, Agoi F, Mahoney MR (2017). Utilization of health services in a resource-limited rural area in Kenya: prevalence and associated household-level factors. PLoS One.

[7] Tanahashi T (1978). Health service coverage and its evaluation. Bull World Health Organ.

[8] Li YN, Nong DX, Wei B (2016). The impact of predisposing, enabling, and need factors in utilization of health services among rural residents in Guangxi, China. BMC Health Serv Res.

[9] Yang G, Wang Y, Zeng Y (2013). Rapid health transition in China, 1990-2010: findings from the global burden of disease study 2010. Lancet..

[10] Jiang Y, Zheng H, Zhao T (2019). Socioeconomic status and morbidity rate inequality in China: based on NHSS and CHARLS data. Int J Environ Res Public Health.

[11] Center for Health Statistics and Information, the Ministry of Health of China (2005). Summary of the report on the 3rd national health services survey analysis. Chinese Hospitals.

[12] National Health and Family Planning Commission of China, scheme of fifth National Health Se- rvice survey of China (in Chinese). Available at: http://www.nhc.gov.cn/mohwsbwstjxxzx/s8211/201610/9f109ff40e9346fca76dd82cecf419ce.shtml. Accessed 26 Oct 2016.

[13] Li A, Shi Y, Yang X, Wang Z (2019). Effect of critical illness insurance on household catastrophic health expenditure: the latest evidence from the National Health Service Survey in China. Int J Environ Res Public Health.

[14] Li X, Lu J, Hu S (2017). The primary health-care system in China. Lancet..

[15] Meng Q, Xu L, Zhang Y (2012). Trends in access to health services and financial protection in China between 2003 and 2011: a cross-sectional study [published correction appears in lancet. 2012;380(9845):888. Lancet..

[16] Huisman M, Kunst AE, Mackenbach JP (2003). Socioeconomic inequalities in morbidity among the elderly; a European overview. Soc Sci Med.

[17] Massamba VK, Talbot D, Milot A (2019). Assessment of the healthy worker survivor effect in the relationship between psychosocial work-related factors and hypertension. Occup Environ Med.

[18] Zhai F, Wang H, Du S (2009). Prospective study on nutrition transition in China. Nutr Rev.

[19] Yin M, Augustin B, Fu Z (2016). Geographic distributions in hypertension diagnosis, measurement, prevalence, awareness, treatment and control rates among middle-aged and older adults in China. Sci Rep.

[20] Wang Z, Chen Z, Zhang L (2018). Status of hypertension in China: results from the China hypertension survey, 2012-2015. Circulation..

[21] Hankivsky O (2012). Women's health, men's health, and gender and health: implications of intersectionality. Soc Sci Med.

[22] Snow RC, Laski L, Mutumba M (2015). Sexual and reproductive health: progress and outstanding needs. Glob Public Health.

[23] Ruggieri A, Anticoli S, D'Ambrosio A (2016). The influence of sex and gender on immunity, infection and vaccination. Ann Ist Super Sanita.

[24] Wu C, Smit E, Xue QL (2017). Prevalence and correlates of frailty among community-dwelling Chinese older adults: the China health and retirement longitudinal study. J Gerontol A Biol Sci Med Sci.

[25] Blümel JE, Arteaga E (2017). The risks of avoiding hormone replacement therapy during menopause. Rev Med Chil.

[26] Jing S, Yin A, Shi L, Liu J (2013). Whether new cooperative Mmedical schemes reduce the economic burden of chronic disease in rural China. PLoS One.

[27] Sun Q, Liu X, Meng Q (2009). Evaluating the financial protection of patients with chronic disease by health insurance in rural China. Int J Equity Health.

[28] Tian D, Sun L, Zhang L (2016). Large urban-rural disparity in the severity of two-week illness: updated results based on the first health service survey of Hunan Province, China. Int J Equity Health.

[29] Cramer RJ, Ireland JL, Hartley V, et al. Coping, mental health, and subjective well-being among mental health staff working in secure forensic psychiatric settings: results from a workplace health assessment. Psychol Serv. 2019. 10.1037/ser0000354. 10.1037/ser0000354 31008626

