# Supplementary material for: Illness prevalence rate in Tibet, China: data from the 2018 National Health Service Survey
Source: BMC Public Health. 2020 Jun 18;20:955. doi: 10.1186/s12889-020-08960-7 (PMC7302388; doi:10.1186/s12889-020-08960-7)
Supplement: Supplementary file 1 — Additional file 1. The classifications of diseases in the Sixth National Health Service Survey, 2018. [file 12889_2020_8960_MOESM1_ESM.pdf]

Additonal file 1

The classifications of diseases in the Sixth National Health Service Survey，2018

Disease

A. **Infectious diseases:** typhoid and paratyphoid; bacterial food poisoning; diarrhea; hepatitis A; other intestinal infections; tuberculosis; tetanus; sepsis, measles, epidemic B encephalitis, epidemic hemorrhagic fever, hepatitis B, leptospirosis, atypical pneumonia, other non-intestinal infectious diseases

B. **Parasitic disease:**malaria, schistosomiasis, other parasitic diseases

C. **malignant tumor:** nasopharyngeal malignancy, esophageal malignancy, gastric malignancy, colon malignancy, rectal and anal malignancy, liver malignancy, pancreatic malignancy, tracheal, bronchial and pulmonary malignancy, breast malignancy, cervical malignancy, leukemia, other malignancy

D. **Benign, in situ and dynamically indeterminate tumors:** benign tumors of the uterus, benign tumors of the brain, other benign tumors, in situ tumors, indeterminate or dynamically indeterminate tumors

E. **Endocrine, nutritional and metabolic diseases and immune diseases:** hyperthyroidism, diabetes, nutritional deficiencies or deficiencies, rickets, obesity and other nutritional excesses, others

F. **Diseases of the blood and hematopoietic organs:** anemia, other diseases of the blood and hematopoietic organs

G. **Psychosis:** organic psychosis of old age, schizophrenia, depression, other mental disorders

H. **Neurological disorders:** meningitis, epilepsy, acute infectious polyneuritis, Parkinson's disease, other neurological disorders

I. **Eye and appendage diseases:** glaucoma, cataract, corneal diseases, other eye and appendage diseases

J. **Ear and mastoid diseases:** otitis media and mastoiditis, other ear and mastoid diseases

K. **Circulatory diseases:** acute rheumatic fever, chronic rheumatic heart disease, angina pectoris, acute myocardial infarction, other ischemic heart disease, pulmonary heart disease, other types of heart disease, hypertension, cerebrovascular disease, lower limb varicose veins, other circulatory diseases

L. **Respiratory diseases:** acute nasopharyngitis (common cold), acute upper respiratory tract

infections such as pharynx, larynx, tonsil and trachea, influenza, pneumonia, chronic pharynx, laryngitis, emphysema, other chronic obstructive pulmonary disease (COPD with chronic branches), asthma, other respiratory diseases (including acute lower respiratory tract infections)

M. **Diseases of the digestive system:** dental diseases, other diseases of the oral or salivary glands and jaws, acute and chronic gastroenteritis, peptic ulcers, diseases of the appendix, abdominal hernia, intestinal obstruction, chronic liver disease and cirrhosis, gallstones and cholecystitis, other diseases of the digestive system

N. **Urogenital diseases:** nephritis and nephropathy, pyelositis, urological calculus, other urological diseases, prostatic hyperplasia and inflammation, other diseases of the male reproductive organs, breast diseases, salpingitis and ovaritis, uterine and vaginal prolapse, other diseases of the female reproductive organs

O. **Pregnancy, childbirth and puerperial complications:** spontaneous abortion, induced abortion, pregnancy and childbirth bleeding, pregnancy hypertension syndrome, normal childbirth, obstructed childbirth, puerperial complications, other pregnancy and puerperial complications

P. **Skin and subcutaneous tissue diseases:** carbuncle and furuncle, dermatitis, other skin and subcutaneous tissue diseases

Q. **Musculoskeletal and connective tissue diseases:** rheumatoid arthritis, disc disease, osteomyelitis and other motor diseases

R. **Congenital abnormality:** congenital heart disease, other congenital abnormality

S. **Perinatal conditions:** premature and immature infants, birth injuries, fetal and neonatal asphyxia, neonatal tetanus, other neonatal diseases

T. **Injuries and poisoning:** fractures, dislocations, sprains and strains, intracranial and internal injuries (including nerves), open injuries and vascular injuries, burns, toxic and toxic effects, other injuries and poisoning

U. ***others:** pregnancy monitoring, sterilization, hospitalization for special treatment, examination of individuals and populations, other reasons

V. **Signs, symptoms and unclear conditions**

*: not a disease
